# Data saves lives: bottom-up, professionally-led endorsement would increase the chance of success

**DOI:** 10.3399/bjgp22X720965

**Published:** 2022-10-28

**Authors:** Simon de Lusignan, Meredith Leston, Margaret Ikpoh, Gary Howsam

**Affiliations:** Nuffield Department of Primary Care Health Sciences, University of Oxford, Oxford.; Nuffield Department of Primary Care Health Sciences, University of Oxford, Oxford.; Royal College of General Practitioners, London.; Royal College of General Practitioners, London.

## NEW INITIATIVES IN THE ENGLISH NHS TO INVEST MORE IN HEALTH DATA

We welcome the publication of the Goldacre Report, *‘Better, broader, safer: using health data for research and analysis’*, and the Secretary of State for Health and Social Care’s ‘Data saves lives’ initiative.^1^^,^^2^ The UK Government has also announced a £200 million investment to support these new strategies and their associated health data repositories.^2^ However, these developments are presented as technical transactions. Greater health community-wide ownership and bottom-up endorsement of data sharing by known and trusted healthcare professionals would increase their chance of success.

## BENEFITS AND POTENTIAL OF ROUTINE DATA SHOWN ACROSS THE PANDEMIC

Routine health data accelerated our understanding of COVID-19 therapies and the impact of vaccination. One important enabler was the temporary relaxation of data sharing restrictions under emergency regulations that occurred during the pandemic; and subsequently withdrawn in July 2022.^3^ It is of note that there was no widespread campaigning or objections to this extended sharing and use of health data among practitioners or the public. Another pandemic success was the acceleration of digital maturity in UK health care; there was much more reliable cross-health service sharing of test results and vaccination data than we see with seasonal influenza, for example. Without effort and sustained investment, as proposed in these initiatives, we risk losing much of this progress.

## STRENGTHS AND LIMITATIONS IN UK HEALTH DATA ECOSYSTEM

A data ecosystem is the complex series of processes that draws together data from a range of disparate but functioning health data systems to enable their analysis and use.^4^ The UK has one of the most successful health data ecosystems globally. For example, primary care, hospital, and death data are frequently linked together to monitor treatment effectiveness.^5^ However, like many others, our data ecosystem is becoming increasingly complex as new apps are added; of concern are those that integrate poorly with primary care computerised medical record (CMR) systems. It is likely this complexity will grow and our future plans need to take account of this.

Many of these limitations are described in the Goldacre Report; however, public mistrust in technology (as opposed to in people) and inconsistencies in health data quality could have been elaborated on further and may undermine many of the recommendations made if left unaddressed.

## TRUSTED RESEARCH ENVIRONMENTS

The role of so-called Trusted Research Environments (TREs) is emphasised throughout the report. TREs are highly secure data environments that provide remote access to health data to approved parties for approved purposes. A small number of TREs are planned to provide rich repositories of both biological and medical record data. Though their use within health care is under development, the aspiration is that they promote transparency, drive innovation within health, and facilitate open working. Specific TRE owners will prevent overcrowding and any duplication of efforts or confusion over who owns what among researchers. Their transparency should ensure they are not ‘black boxes’, as has been a major criticism of past technological reforms in the NHS.

## CENTRALISM OR A FEDERATED MODEL FOR TREs

Centralism does not always achieve more — especially in the context of innovation. The risks are that streamlining decision-making in this way may meet the needs of some groups but not others, and that the expertise of specialist teams may be lost as a result of this standardisation. It is all too easy to create bottlenecks that block progress. A federated TRE model underpinned by converging standards but mindful of the value of expert groups could be the way forward; examples are shown in Table 1. Such a federated approach could facilitate the development of the ‘talent pipeline’ of in-house analysts that Goldacre describes while ensuring deep and domain-specific expertise remains.

**Table 1.** UK data sources, from oldest to most recently established, that have contributed to COVID-19 research and/or surveillance and could contribute to a federated trusted research environment

**Database acronym**	**Full name or database**	**Coverage**	**Size (patients)**	**Established**	**Role in COVID-19**
RCGP RSC	Oxford–Royal College of General Practitioners’ Research and Surveillance Centre	England (historically Wales)	>18 m	1957 1967: sentinel surveillance	Sentinel surveillance and research
CPRD	Clinical Practice Research Datalink	UK	>60 m, including 16 m currently registered	1987 1993: known as General Practice Research Database 2012: changed to CPRD	Risk groups and research
QRsearch	N/A	UK	>35 m	2003	Risk groups and research
EAVE II	Early Assessment of Vaccine and anti-viral Effectiveness	Scotland	>5.4 m	2009	Sentinel surveillance and research
SAIL Databank	Secure Anonymised Information Linkage Databank	Wales	>5 m records	2006	Sentinel surveillance and research
OpenSAFELY	N/A	UK	>58 m	2020	Risk groups and research

*m = million. N/A = not applicable.*

## IMPROVING DATA QUALITY AND UNDERSTANDING THE CONTEXT OF DATA RECORDING

TREs may not be a panacea for managing and analysing health data, as great, if not greater, priority should be improving data quality and understanding the context of data recording to ensure its correct interpretation. CMRs are a complex mix of registration data, coded clinical data, and free-text. Realising the full benefits of routine data will require improvements to clinical coding. The value of clinician coding, particularly of presenting problem, is that it provides an experienced clinician’s view of the likely underlying diagnosis. Pay- for- performance schemes, like the Quality and Outcomes Framework, drive the use of specific disease codes and the improvement of data quality, but such incentives cannot be made universal. Much more use could be made of free-text if we could apply privacy protecting mechanisms to its analysis.

## TRUST

UK primary care professionals are trusted by their patients^6^ and show professionalism in facilitating data sharing.^7^ The Goldacre Report could have included more about engaging practices and providing tangible benefits to clinicians and their patients. There is scope to engage, even co-design, with practitioners, patients, and service users, for example.^8^

## UNINTENDED CONSEQUENCES

Top-down changes can cause unintentional disruptions and render a complex ecosystem dysfunctional. Too often such change fails to consider the need for local engagement. Trust is hard won, easily lost, and must be cultivated between people. The difficulties over health data being used to support wider use-cases, such as asylum seeking, reflects this.^9^

## IN SUMMARY

The Goldacre Report and the ‘Data saves lives’ initiative are both welcome and timely. As Goldacre puts it best, *‘it is our collective duty to make this work’*.^1^

We recommend strengthening this report in three important areas. First, by federating existing data expertise into these national TREs. Second, by finding ways that higher data quality can be used to reduce bureaucracy in primary care. Finally, by ensuring that the benefit–risks of data sharing are supported by communications from trusted clinicians. In doing so, we can be sure to strengthen the successful but sensitive health data ecosystem of the UK and minimise the risk of disruption.

## References

[1] Goldacre B, Morley J, Hamilton N (2022). Better, broader, safer: using health data for research and analysis.

[2] Javid S, Department of Health and Social Care (2022). Data saves lives: reshaping health and social care with data. https://www.gov.uk/government/speeches/data-saves-lives-reshaping-health-and-social-care-with-data.

[3] Department of Health and Social Care (2022). [withdrawn] Coronavirus (COVID-19): notice under regulation 3(4) of the Health Service (Control of Patient Information) Regulations 2002 – general. https://www.gov.uk/government/publications/coronavirus-covid-19-notification-of-data-controllers-to-share-information/coronavirus-covid-19-notice-under-regulation-34-of-the-health-service-control-of-patient-information-regulations-2002-general--2.

[4] Barker W, Johnson C (2021). The ecosystem of apps and software integrated with certified health information technology. J Am Med Inform Assoc.

[5] Agrawal U, Bedston S, McCowan C (2022). Severe COVID-19 outcomes post full vaccination of primary schedule and boosters: A meta-analysis of national prospective cohort studies of 30 million individuals in England, Northern Ireland, Scotland and Wales. Lancet.

[6] Croker JE, Swancutt DR, Roberts MJ (2013). Factors affecting patients’ trust and confidence in GPs: evidence from the English national GP patient survey. BMJ Open.

[7] de Lusignan S, Chan T, Theadom A, Dhoul N (2007). The roles of policy and professionalism in the protection of processed clinical data: a literature review. Int J Med Inform.

[8] Hutchings R, Edwards N, Scobie S (2021). Fit for the future: what can the NHS learn about digital health care from other European countries?.

[9] Gulland A (2017). Handing NHS data to the Home Office. BMJ.

